# Large hemorrhagic pericardial effusion with cardiac tamponade in a 16-year-old adolescent in an endemic area of tuberculosis: a case report

**DOI:** 10.11604/pamj.2021.40.117.10551

**Published:** 2021-10-22

**Authors:** Clovis Nkoke, Christelle Makoge, Denis Tewafeu, Cyrille Nkouonlack, Charifa Njoya, Ines Nepetsoun, Engelbert Bain Luchuo, Ahmadou Musa Jingi

**Affiliations:** 1 Faculty of Medicine and Biomedical Sciences, Department of Internal Medicine, University of Yaoundé I, Yaoundé, Cameroon,; 2 Buea Regional Hospital, Buea, South West Region, Cameroon,; 3 Center for Population Studies and Health Promotion, Yaoundé, Cameroon

**Keywords:** Pericardial effusion, cardiac tamponade, tuberculosis, case report

## Abstract

Pericardial effusion complicated by cardiac tamponade is a medical emergency. Large pericardial effusion and tamponade are rare in childhood. Tuberculosis remains a major cause of pericardial effusion in endemic areas. A 16-year-old adolescent with no significant past history was admitted to the medical unit of the Buea Regional hospital in the South West region of Cameroon for heart failure after presenting with abdominal distension, shortness of breath and fever of two weeks duration. Echocardiographic study during admission revealed a large pericardial effusion (27mm in thickness) with echocardiographic signs of tamponade. Echocardiographic guided pericardiocentesis was performed through a sub-xiphoid route and about 500 cc of heavily stained blood fluid that was not coagulating was drained. Pericardial fluid analysis for acid fast bacilli was negative. There was no evidence of malignancy. A strong suspicion of tuberculosis was made and he was started on anti-tuberculosis medications for presumptive hemorrhagic tuberculous pericarditis. Patient was asymptomatic during follow up and repeat echocardiographic examinations showed no re-accumulation of pericardial fluid. Tuberculosis should be considered as the etiology of pericardial effusion in endemic areas although the identification of mycobacterium is challenging in these settings.

## Introduction

Pericardial effusion is a relatively common finding in clinical practice. It can be complicated by cardiac tamponade which is a medical emergency but large pericardial effusion and tamponade are rare in childhood. The etiologies are diverse and may include trauma; infection, malignancy endocrine or radiation [1]. Tuberculous pericarditis continues to have a high incidence in developing countries; it is a frequent cause of hemorrhagic effusion in endemic areas. It is the most common cause of pericarditis in Africa and other countries in which tuberculosis remains a major public health problem [2]. In one series from the Western Cape Province of South Africa, tuberculous pericarditis accounted for 69.5% (162 of 233) of cases referred for diagnostic pericardiocentesis [3].The echocardiogram is the most available and reliable technique in order to verify the presence and the amount of a pericardial effusion; in addition, the echocardiogram provides valuable information for evaluation of hemodynamic consequences. Diagnosis and treatment of pericardial effusions is challenging in resource limited settings.

## Patient and observation

**Patient information**: a 16-year-old boy who was a student, was admitted to the medical unit of the Buea regional Hospital after he presented with a two week history of gradually worsening shortness of breath and abdominal distension, non-productive cough, loss of appetite and weight loss. These became associated with fever three days prior to admission. There were no night sweats, and no sputum. There was no history of trauma.

**Clinical findings**: on physical examination, his temperature was 36.7°C, respiratory rate was 24 breaths/minute, heart rate 98/minute; blood pressure 87/71mmHg. Cardiovascular examination was remarkable for distended neck veins, hepatojugular reflux and pulsus paradoxus. There was no lower extremity edema. The heart sounds were not muffled. There was no pericardial friction rub. Lung examination revealed bilateral pleural effusions. Abdominal examination revealed ascites and a tender hepatomegaly. There were neither lymphadenopathies nor palpable masses to suggest malignancy.

**Figure 1:**
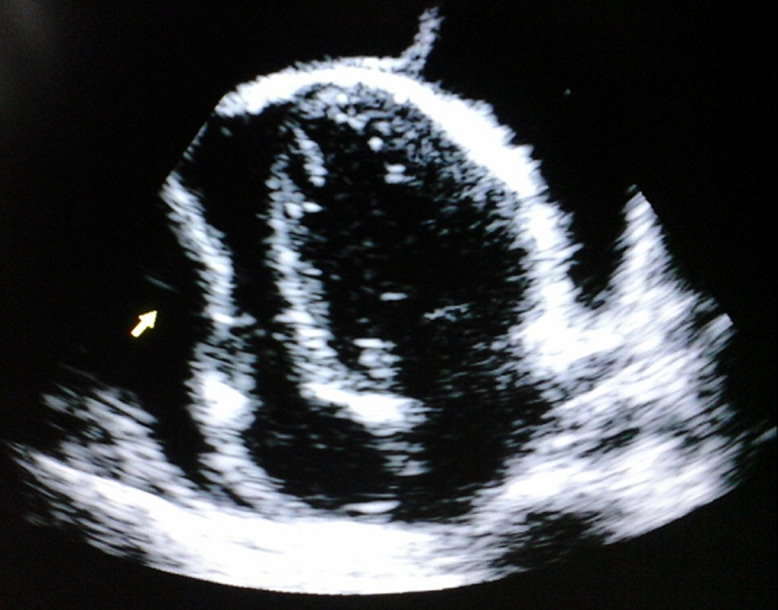
apical four chambers view showing a circumferential pericardial effusion with collapse of the right ventricle in diastole (white arrow); the surface of the heart has a shaggy appearance, with frond-like structures extending to the parietal pericardium, this appearance is typical of tuberculous pericardial effusion

**Diagnostic assessment**: the chest x-ray showed an enlarged cardiac silhouette with bilateral pleural effusions. The HIV test was negative. The C-reactive protein was 24mg/l, erythrocyte sedimentation rate equal 45mm. Echocardiogram showed a large circumferential pericardial effusion with right ventricular diastolic collapse and marked variation in tricuspid and mitral inflow suggestive of cardiac tamponade (Figure 1). Echocardiographic guided pericardiocentesis was performed through the sub-xiphoid route and about 500cc of hemorrhagic fluid was drained which improved his symptoms. Fluid analysis was consistent with an exudative effusion. Acid fast bacilli stain was negative. Extensive evaluation of the etiology was not performed because of limited resources in this rural hospital. A strong suspicion of tuberculosis was made and a presumptive treatment for hemorrhagic tuberculous pericarditis was started.

**Therapeutic intervention**: pericardiocentesis was performed and the patient was placed on anti-tuberculosis medications.

**Figure 2:**
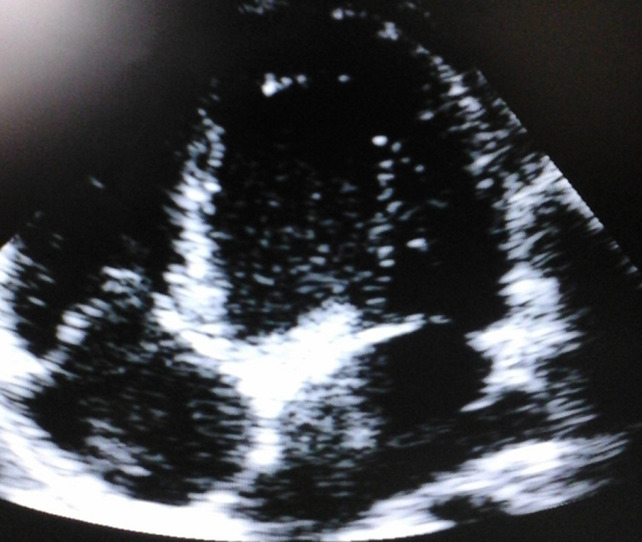
apical four chambers view showing the absence of re-accumulation of pericardial effusion

**Follow-up and outcomes**: a follow-up echocardiography after two weeks did not show re-accumulation of the pericardial fluid (Figure 2). Clinically, the dyspnea had regressed. There were no signs of right sided heart failure and no signs of cardiac tamponade. He has no adverse effects from the medications.

**Patient perspective**: during the time he was hospitalized and after the treatment, the patient and his family were delighted with the care he received and was optimistic about the outcome of his condition.

**Informed consent**: the patient and the family were informed about the case report, why the case was peculiar and the authors' interest in publishing his case. The family willingly gave informed consent to allow the authors to use his echo images for this case report.

**Patient's consent**: informed consent was obtained from the family for us to use the echo images and to publish the case.

## Discussion

We have reported the case of a-16-year-old boy with a large hemorrhagic pericardial effusion with tamponade treated presumptively as tuberculous pericardial effusion in a new cardiac service in a rural setting in Cameroon. We present this case due to its rarity in childhood and to highlight the diagnostic and therapeutic challenges in rural settings. Hemorrhagic pericardial effusion can be caused by malignancy, trauma, bleeding diathesis, tuberculosis and uremia [4]. Although hemorrhagic pericardial effusion most often suggests a malignancy, such an event is not rare in tuberculosis especially in those countries where tuberculosis is endemic where it constitutes an important etiology of hemorrhagic pericardial effusion [2,3,5]. The pericardial fluid is blood stained in 80% of cases of tuberculous pericarditis. Tuberculosis is detected in 1-2% of cases of acute pericarditis in developing countries and about 7% of these cases present with cardiac tamponade. Mortality ranges from 14-40% [5].

The diagnosis of tuberculous pericarditis includes identification of Mycobacterium tuberculosis from the pericardial tissue or fluid cultures, histopathologic demonstration of granulomas or acid-fast bacilli in the pericardial tissue and response to specific antituberculosis therapy [6,7]. In our patient, other than response to anti-tuberculosis therapy and absence of mycobacterium on microcopy of pericardial fluid, none of the other diagnostic criteria could be performed in this setting, posing a real challenge for etiologic diagnosis. Mycobacterium tuberculosis is quite difficult to be isolated in pericardial fluid and is present only in one third cases. Hemorrhagic pericardial effusion caused by tuberculosis has been reported in immunocompromised adults and geriatric patients [8] but it is quite rare in immunocompetent children. The limitation in our case was that we did not perform and extensive search for the etiology of the pericardial effusion. As a result the patient was treated presumptively for tuberculous pericardial effusion based on epidemiologic data. However he responded well to anti-tuberculosis therapy.

## Conclusion

Pericardial effusion with cardiac tamponade is a life-threatening medical emergency that requires prompt diagnosis and emergent treatment. Hemorrhagic pericardial effusion is a red flag that justifies a careful search for sinister etiologies, especially malignancy and tuberculosis, as the mortality rate is high if left untreated. But this is not always possible in resource limited settings. However, tuberculosis remains a common etiology in sub-Saharan Africa.
